# Long-term survival among patients with gastrointestinal stromal tumors diagnosed after another malignancy: a SEER population-based study

**DOI:** 10.1186/s12957-020-01868-x

**Published:** 2020-05-06

**Authors:** Chaoyong Shen, Chengshi Wang, Tao He, Zhaolun Cai, Xiaonan Yin, Yuan Yin, Donghao Lu, Bo Zhang, Zongguang Zhou

**Affiliations:** 1 Department of Gastrointestinal Surgery, West China Hospital, Sichuan University, Chengdu, 610041 Sichuan China; 2 Clinical Research Center for Breast Diseases, Laboratory of Molecular Diagnosis of Cancer, and Department of Medical Oncology, West China Hospital, Sichuan University, Chengdu, Sichuan China; 3 Department of Breast Surgery, West China School of Medicine/West China Hospital, Sichuan University, Chengdu, China; 4 Department of Medical Epidemiology and Biostatistics, Karolinska Institutet, Stockholm, Sweden; 5 Laboratory of Molecular Diagnosis of Cancer, Clinical Research Center for Breast, West China Hospital, Sichuan University, Chengdu, Sichuan China; 6 Institute of Digestive Surgery and State Key Laboratory of Biotherapy, West China Hospital, Sichuan University, Chengdu, 610041 Sichuan China

**Keywords:** Gastrointestinal stromal tumors, Population-based, Second malignant neoplasms, Prognosis, SEER

## Abstract

**Background:**

To explore overall survival (OS) and GISTs-specific survival (GSS) among cancer survivors developing a second primary gastrointestinal stromal tumors (GISTs).

**Methods:**

We conducted a cohort study, where patients with GISTs after another malignancy (AM-GISTs, *n* = 851) and those with only GISTs (GISTs-1, *n* = 7660) were identified from the Surveillance, Epidemiology, and End Results registries (1988–2016). Clinicopathologic characteristics and survival were compared between the two groups.

**Results:**

The most commonly diagnosed first primary malignancy was prostate cancer (27.7%), followed by breast cancer (16.2%). OS among AM-GISTs was significantly inferior to that of GISTs-1; 10-year OS was 40.3% vs. 50.0%, (*p* < 0.001). A contrary finding was observed for GSS (10-year GSS 68.9% vs. 61.8%, *p* = 0.002). In the AM-GISTs group, a total of 338 patients died, of which 26.0% died of their initial cancer and 40.8% died of GISTs. Independent of demographics and clinicopathological characteristics, mortality from GISTs among AM-GISTs patients was decreased compared with their GISTs-1 counterparts (HR, 0.71; 95% CI, 0.59–0.84; *p* < 0.001), whereas OS was inferior among AM-GISTs (HR, 1.11; 95% CI, 0.99–1.25; *p* = 0.085).

**Conclusions:**

AM-GISTs patients have decreased risk of dying from GISTs compared with GIST-1. Although another malignancy history does not seemingly affect OS for GISTs patients, clinical treatment of such patients should be cautious.

## Introduction

Gastrointestinal stromal tumors (GISTs), which originate from the interstitial cells of Cajal or its precursor, are a group of mesenchymal neoplasms with a varying malignancy potential [1]. With an obviously increasing incidence in the past two decades, GISTs are still uncommon, accounting for 3% of all gastrointestinal tumors and approximately 20% of soft sarcomas [2, 3]. The majority of GISTs develop sporadically, and activating mutations of *KIT* and *PDGFRA* occur in the majority of GISTs, which play a central role in GISTs occurrence and development [4]. The introduction of imatinib mesylate has revolutionized the treatment of GISTs, and its prognosis has been significantly improved in recent years.

Advances in the screening, treatment, and management of cancers have led to significant increase in survivor over the past few decades. From 1991 to 2016, the total cancer death rate continued to decline by 27%, which results in an increasing number of cancer survivors in the USA [5]. In such a large population, many cancer survivors are at increased risk of developing other malignancies, due to shared cancer treatment, common etiological exposures, and intrinsic genetic mutations of the first primary ones [6, 7]. In parallel, the lifetime risk of developing a second primary malignancy may be as high as 8~34% [8, 9]. There is a large body of literature describing the risk of cancer survivors suffering from a second primary malignancy, such as those with Hodgkin lymphoma (HL) [10–12], breast cancer [13], and thyroid cancer [14]. In recent years, GISTs occur asynchronously with other malignancies during their clinical course is relatively common [15, 16]. Albeit the GISTs as a second primary malignancy is also increasingly diagnosed, but the prognosis is poorly described.

Clinical decision-making for GISTs patients after another malignancy (AM-GISTs), however, has been challenging due to limited information on prognosis available. Most investigations consist of single-institution series or based on small samples (range, 1 to 97 patients) [17–20]. No large-scale, population-based study has comprehensively examined long-term survival among patients with AM-GISTs, taking into account demographic and treatment-associated variables. It is unclear that the most common first primary malignancy sites in those patients yet. Additionally, this is largely unknown whether AM-GISTs have a different invasiveness when comparing to GISTs as the only malignancy. As such, we have come to realize that it is necessary to address overall survival (OS) and GISTs-specific survival (GSS) for patients with AM-GISTs.

We therefore identified patients with GISTs diagnosis after another malignancy by utilizing the well-established Surveillance, Epidemiology, and End Results (SEER) database, to explore the OS and GSS. Cancer-related variables and clinicopathologic characteristics were analyzed to assess their impact on prognosis. This may help to better understand appropriate long-term surveillance strategies and highlight the need for future efforts at prevention and intervention.

## Materials and methods

### Patients

All patients diagnosed with histologically confirmed GISTs as a second primary neoplasm after another malignancy were identified in population-based registries of the SEER-18 Program (1988–2016). The National Cancer Institute’s SEER database is a comprehensive database that compiles information regarding cancer incidence and survival and is approximately to encompass 34.6% of the US population (http://seer.cancer.gov/about/ overview.html). We have been licensed by SEER to access the research data (reference number 10185-Nov 2018). Due to the strict register-based nature of the study, informed consent was waived. Moreover, the study was exempted from Institutional Review Board approval, in view of the SEER’s use of unidentifiable patient information.

**Fig. 1:**
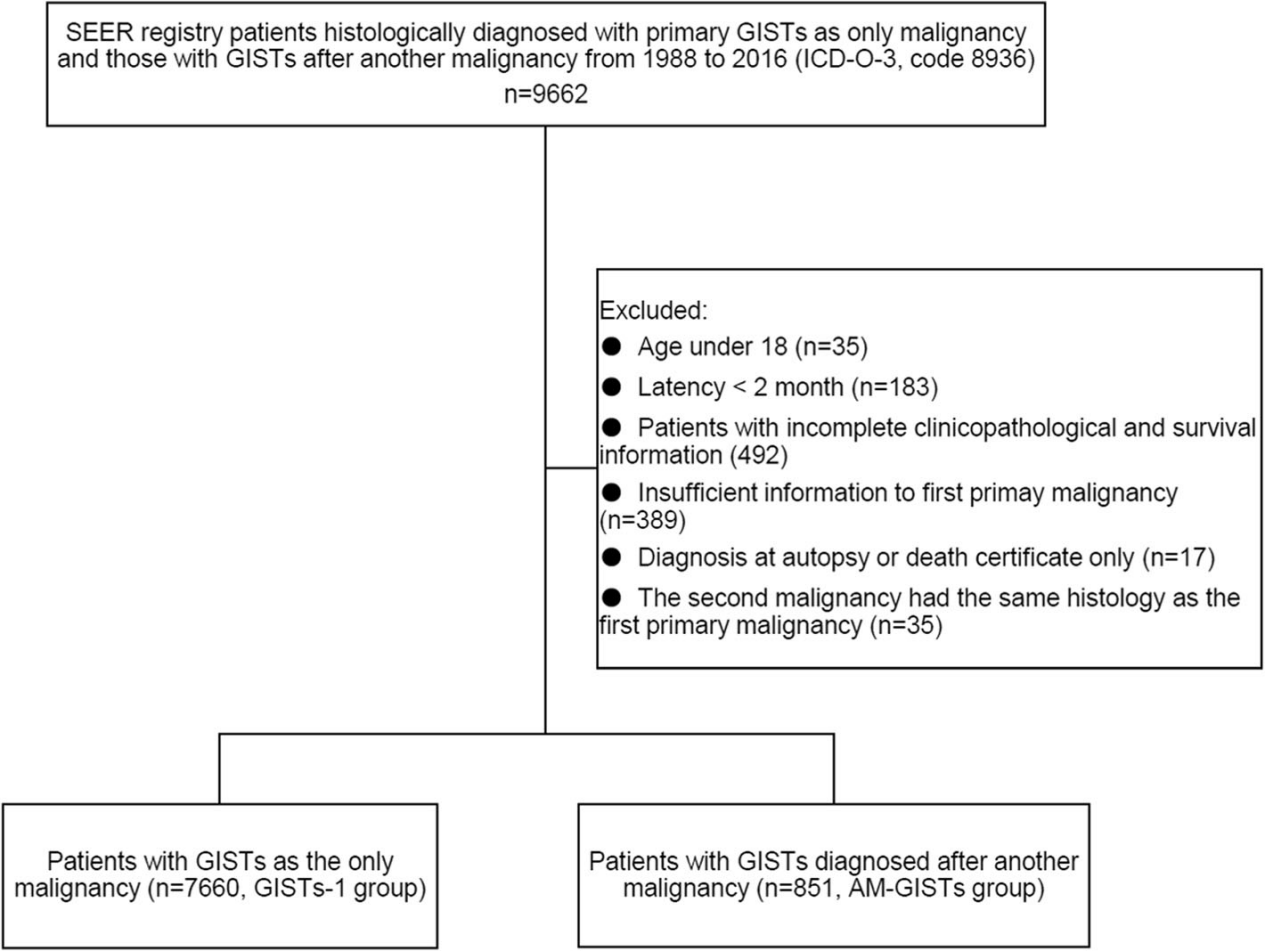
The flow diagram of patient selection

The National Cancer Institute SEER*Stat software (Version 8.3.5) was used to identify patients. GISTs were identified by the tumor site, sequence number, and the histological code (International Classification of Diseases for Oncology, Third Edition [ICD-O-3], code 8936). Patients were divided into two groups as mentioned in previous studies [18, 21]: GISTs-1 group (GISTs as the only malignancy) and AM-GISTs group (patients with GISTs diagnosed after another malignancy and those with ≥ 3 pathologic diagnosis of cancers were not included). The exclusion criteria were as follows: (1) those with a diagnosis at autopsy or death certificate only, (2) the second malignant tumor had the same histology as the first malignancy, (3) the second primary malignancy was diagnosed within 2 months of the first malignancy (as used in SEER to rule out synchronous primary cancer), (4) patients aged < 18 years old, and (5) patients with missing/incomplete clinicopathological information (survival data, details of the first primary malignancy). Totally, we identified 8511 patients with primary gastrointestinal stromal tumors, including 7660 GISTs-1 and 851 AM-GISTs. The flow diagram of data selection is shown in Fig. 1.

### Primary outcome and covariates of interest

The primary outcome of interest was GSS and OS as a secondary outcome. Patient demographics included sex, age at diagnosis, ethnicity, marital status, year of diagnosis, education, and household income status (as determined from census data). Tumor characteristics included primary site, tumor size, grade, and SEER stages based on the SEER Summary Staging Manual. Tumor size and age as continuous variables which were transformed into categorical variables according to recognized cut-off values. All patients were followed from cancer diagnosis to December 31, 2016, or death, whichever came first. OS was estimated by the Kaplan-Meier method, with survival times measured from date of GISTs diagnosis until date of death from any cause or last follow-up. GSS was calculated as the interval from initial diagnosis to death because of GISTs or censoring, excluding other causes of death; thus, only death from GISTs was designated as an event.

### Statistical analysis

Calculations were performed with the Statistical Package for the Social Science (SPSS), version 21.0 for Windows (SPSS Inc, Chicago, IL, USA). Numerical data were expressed as mean ± standard deviation or median for quantitative variables analyzed using one-way ANOVA. Continuous variables were first transformed into categorical data, and categories were described as frequencies and percentage and then compared with Chi-square test. Cumulative survival was determined by the Kaplan-Meier method, and the statistical significance was determined by the log-rank test. Next, we conducted the cumulative mortality rates by cause of death due to GIST for patients with AM-GISTs and GISTs-1 up to 10 years after cancer diagnosis using a competing risk model. We then estimated hazard ratios (HR) using multivariable Cox proportional hazards models that incorporated our covariates of interest (tumor site, tumor grade, sex, marital status, age, race, and SEER stage, etc.), to explore independent prognostic values for GISTs-specific and overall survival. Hazard ratios and 95% confidence intervals were assessed for each variables. All *p* values were two-sided, with *p* < 0.05 indicated statistically significant.

## Results

### Patient and tumor characteristics

**Fig. 2:**
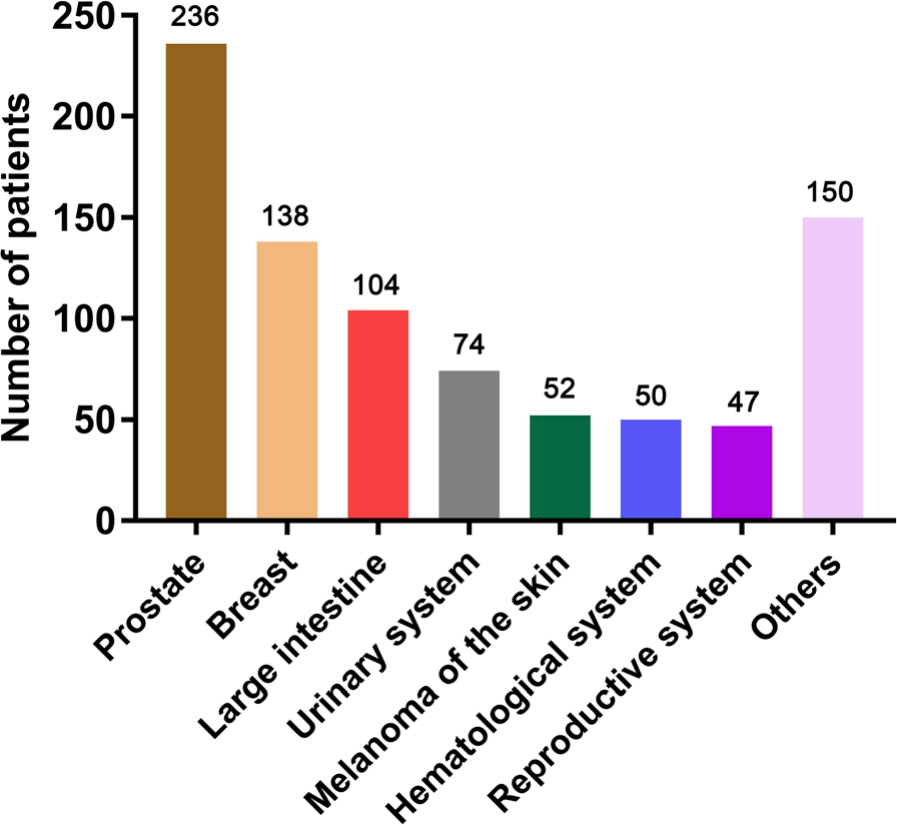
Sites of most common first primary malignancy in the AM-GISTs group. GISTs indicate gastrointestinal stromal tumors

Of 8511 patients with histologically confirmed GISTs in this study, 851 had AM-GISTs, and 7660 had GISTs-1, with a median follow-up of 44 months (range, 0.5~329 months). Figure 2 displays the distribution of the first primary malignancy sites. The most commonly diagnosed first primary malignancy was prostate cancer (*n* = 236, 27.7%), followed by breast cancer (*n* = 138, 16.2%), carcinoma of large intestine (*n* = 104, 12.2%), and malignant tumor of urinary system (*n* = 74, 8.7%).

Table 1 outlines demographic and clinicopathologic characteristics of patients with AM-GISTs and GISTs-1 at time of GISTs diagnosis. The age at diagnosis of GISTs ranged from 18 to 101 years, with an average and a median age at diagnosis of 62 and 63 years, respectively. The age at diagnosis was 61.8 ± 14.4 years in the GISTs-1 group, which was younger than that of AM-GISTs group (69.3 ± 11.5 years, *p* < 0.001). AM-GISTs were diagnosed in more recent years and at obviously more advanced ages than GISTs-1 (aged ≥ 66 years, 65.1% vs. 41.6%). Moreover, the proportion of males in AM-GISTs was higher than GISTs-1, with corresponding percentages of 56.5% and 51.6%, respectively (*p* = 0.006). The GISTs stage distribution of AM-GISTs (57.9% localized, 8.7% regional, 15.9% distant) differed significantly from GISTs-1 (52.1% localized, 12.5% regional, 20.9% distant). Patients with AM-GISTs were considerably more likely to have a smaller tumor size than GISTs-1 group (*p* < 0.001). It is worthy to note that lymph node metastases were found in 2.3% of all patients. Additionally, there was no significant difference in median household income, primary site, and grade distribution between patients with AM-GISTs and GISTs-1 (*p* > 0.05). Median latency between diagnoses of first primary another malignancy and GISTs was 4 years, with a range of 2 months to 31 years.

**Table 1:** Patient and tumor characteristics at time of gastrointestinal stromal tumors diagnosis among 8511 patients: a SEER population-based study in US, 1988–2016

Covariates	All patients (*n* = 8511, %)	AM-GISTs (*n* = 851, %)	GISTs-1 (*n* = 7660, %)	p*
**Age at diagnosis, years**				**< 0.001**
18–35	322 (3.8)	7 (0.8)	315 (4.1)	
36–50	1401 (16.5)	44 (5.2)	1357 (17.7)	
51–65	3049 (35.8)	246 (28.9)	2803 (36.6)	
66–80	2694 (31.7)	383 (45.0)	2311 (30.2)	
≥ 80	1045 (12.3)	171 (20.1)	874 (11.4)	
**Sex**				**0.006**
Male	4434 (52.1)	481 (56.5)	3953 (51.6)	
Female	4077 (47.9)	370 (43.5)	3707 (48.4)	
**Race/ethnicity**				**0.005**
White	5755 (67.6)	606 (71.2)	5149 (67.2)	
Black	1561 (18.3)	156 (18.3)	1405 (18.3)	
Other†	1195 (14.0)	89 (10.5)	1106 (14.4)	
**Marital status**				**0.001**
Single	1386 (16.3)	100 (11.8)	1286 (16.8)	
Married/domestic partner	4909 (57.7)	504 (59.2)	4405 (57.5)	
Widowed/separated/divorced	1799 (21.1)	207 (24.3)	1592 (20.8)	
Unknown	417 (4.9)	40 (4.7)	377 (4.9)	
**%< High school education, tertile**				**0.038**
First tertile	2812 (33.0)	307 (36.1)	2505 (32.7)	
Second tertile	3004 (35.3)	305 (35.8)	2699 (35.2)	
Third tertile	2695 (31.7)	239 (28.1)	2456 (32.1)	
**Median household income, tertile**				**0.478**
First tertile	2811 (33.0)	278 (32.7)	2533 (33.1)	
Second tertile	2830 (33.3)	271 (31.8)	2559 (33.4)	
Third tertile	2870 (33.7)	302 (35.5)	2568 (33.5)	
**Year of diagnosis**				**< 0.001**
1988–2002	1139 (13.4)	81 (9.5)	1058 (13.8)	
2003–2016	7372 (86.6)	770 (90.5)	6602 (86.2)	
**Primary site**				**0.309**
Stomach	4960 (58.3)	515 (60.5)	4445 (58.0)	
Small intestine	2329 (27.4)	215 (25.3)	2114 (27.6)	
Other	1222 (14.4)	121 (14.2)	1101 (14.4)	
**Chemotherapy**				**< 0.001**
Yes	3440 (40.4)	282 (33.1)	3158 (41.2)	
No/unknown	5071 (59.6)	569 (66.9)	4502 (58.8)	
**Surgery of primary**				**0.386**
Yes	6566 (77.1)	641 (75.3)	5925 (77.3)	
No	1766 (20.7)	192 (22.6)	1574 (20.5)	
Unknown	179 (2.1)	18 (2.1)	161 (2.1)	
**Lymph node metastasis**				**0.024**
Yes	200 (2.3)	14 (1.6)	186 (2.4)	
No	1966 (23.1)	171 (20.1)	1795 (23.4)	
Unknown	6345 (74.6)	666 (78.3)	5679 (74.1)	
**Tumor size, cm**				**< 0.001**
0–2	602 (7.1)	106 (12.5)	496 (6.5)	
> 2 to ≤ 5	1908 (22.4)	208 (24.4)	1700 (22.2)	
> 5 to ≤ 10	2311 (27.2)	199 (23.4)	2112 (27.6)	
> 10	1933 (22.7)	159 (18.7)	1774 (23.2)	
Unknown	1757 (20.6)	179 (21.0)	1578 (20.6)	
**Grade**				**0.184**
Well differentiated	1077 (12.7)	121 (14.2)	956 (12.5)	
Moderately differentiated	900 (10.6)	104 (12.2)	796 (10.4)	
Poorly differentiated	361 (4.2)	30 (3.5)	331 (4.3)	
Undifferentiated	544 (6.4)	52 (6.1)	492 (6.4)	
Unknown	5629 (66.1)	544 (63.9)	5085 (66.4)	
**SEER stages**				**< 0.001**
Localized	4483 (52.7)	493 (57.9)	3990 (52.1)	
Regional	1033 (12.1)	74 (8.7)	959 (12.5)	
Distant	1739 (20.4)	135 (15.9)	1604 (20.9)	
Unstaged/unknown	1256 (14.8)	149 (17.5)	1107 (14.5)	
**Latency**‡				**NA**
≥ 2 months to 5 years	NA	496 (58.3)	NA	
> 5–10 years	NA	281 (20.9)	NA	
> 10–15 years	NA	103 (12.1)	NA	
> 15–20 years	NA	42 (4.9)	NA	
> 20 years	NA	32 (3.8)	NA	

*GISTs* gastrointestinal stromal tumors, *NA* not applicable (because variable only applies to AM-GISTs group), *SEER* the Surveillance, Epidemiology, and End Results database

*p value was calculated using the chi-square test

†American Indian, Alaskan Native, or Asian/Pacific Islander

‡Latency was calculated as the number of months/years between first primary malignancy diagnosis and GISTs diagnosis

### Details of vital status and cause of death

Table 2 describes the vital status and cause of death in the current study cohort. Of patients with AM-GISTs, 338 (39.7%) were deceased, compared with 2730 (35.6%) of patients in the GISTs-1 group. A total of 71.5% of all deaths can be attributed to either a first or second primary malignancy. Deaths from GISTs were approximately twice as common in the GISTs-1 group versus AM-GISTs group (72.1% vs. 40.8%). For patients with AM-GISTs, 88 (26.0%) died of their initial cancer; causes of death were prostate cancer (*n* = 14), carcinoma of large intestine (*n* = 9), malignant tumor of urinary system (*n* = 9), breast cancer (*n* = 8), hematologic malignancy (*n* = 8), and others (*n* = 40). Cardiovascular/heart disease accounted for a relatively small number (*n* = 307) of deaths, with corresponding percentage of 11.2% and 9.9% in AM-GISTs and GISTs-1 group, respectively. A total of 21.9% of patients in the AM-GISTs died from other noncancer causes, compared with 18.1% of patients in the GISTs-1 group.

**Table 2:** Vital status and cause of death among patients with gastrointestinal stromal tumors after another malignancy (AM-GISTs) and GISTs only (GISTs-1)

	All patients (*n* = 8511, %)	AM-GISTs (*n* = 851, %)	GISTs-1 (*n* = 7660, %)	p*
**Vital Status**				**0.019**
Alive at last follow-up	5443 (71.1)	513 (60.3)	4930 (64.4)	
Dead	3068 (40.1)	338 (39.7)	2730 (35.6)	
**Cause of death**				**0.048**
First cancer	88 (2.9)	88 (26.0)	NA	
GISTs	2105 (68.6)	138 (40.8)	1967 (72.1)	
Cardiovascular/heart disease	307 (10.0)	38 (11.2)	269 (9.9)	
Other noncancer†	568 (18.5)	74 (21.9)	494 (18.1)	

*GISTs* gastrointestinal stromal tumors, *NA* not applicable

*p value was calculated using the chi-square test

†Mainly including cerebrovascular and chronic obstructive pulmonary disease, diabetes mellitus, nephrosis, and Alzheimers et al.

### Survival analysis of AM-GISTs versus GISTs-1 groups

**Fig. 3:**
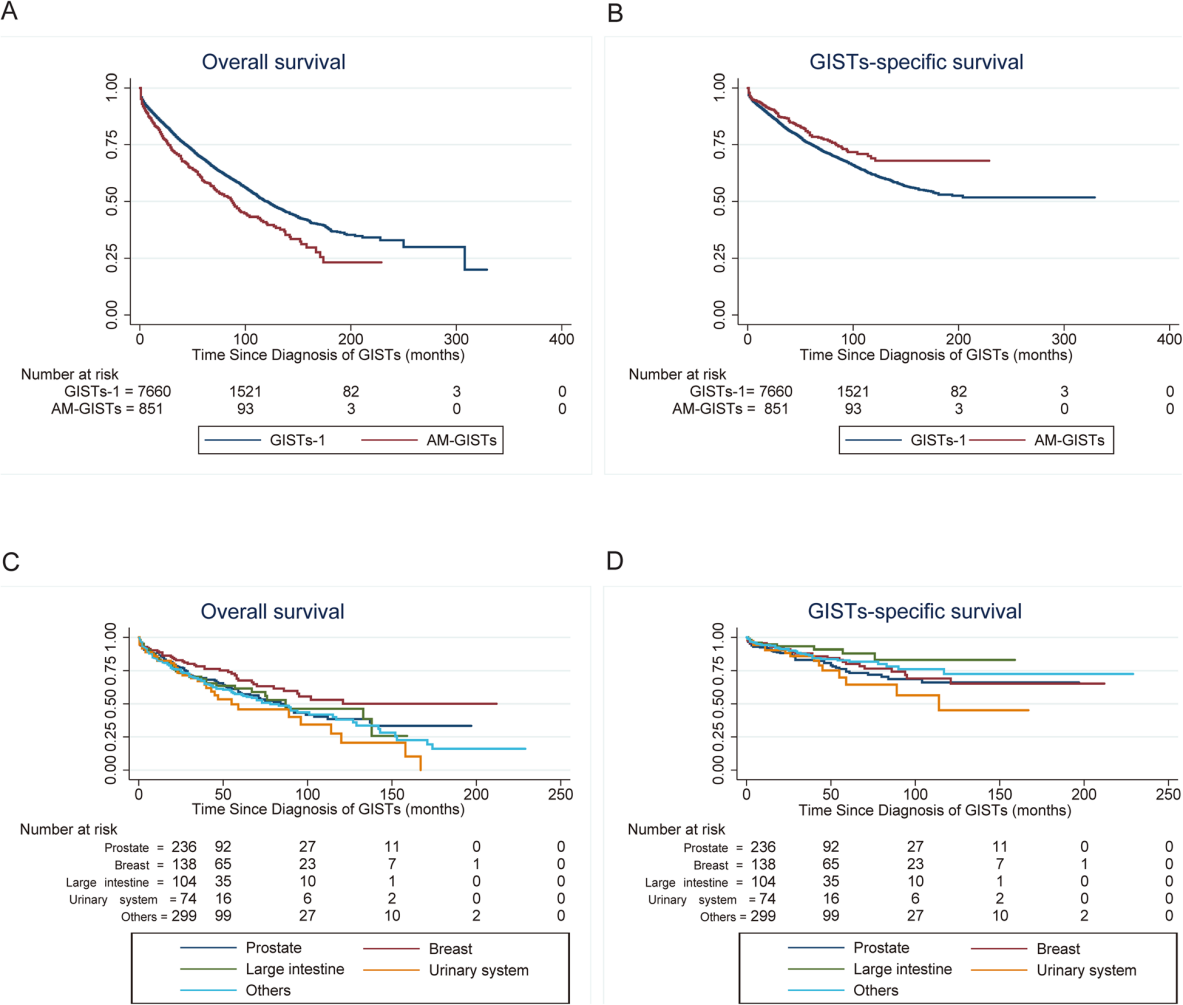
Kaplan-Meier OS and GISTs-specific survival for patients with AM-GISTs and GISTs-1. Differences of OS (**a**) and GISTs-specific survival (**b**) were significant (*p* < 0.001, *p* = 0.002; respectively) between patients with AM-GISTs and GISTs-1. The OS (**c**) and GISTs-specific survival (**d**) grouped by first primary malignancy sites

**Fig. 4:**
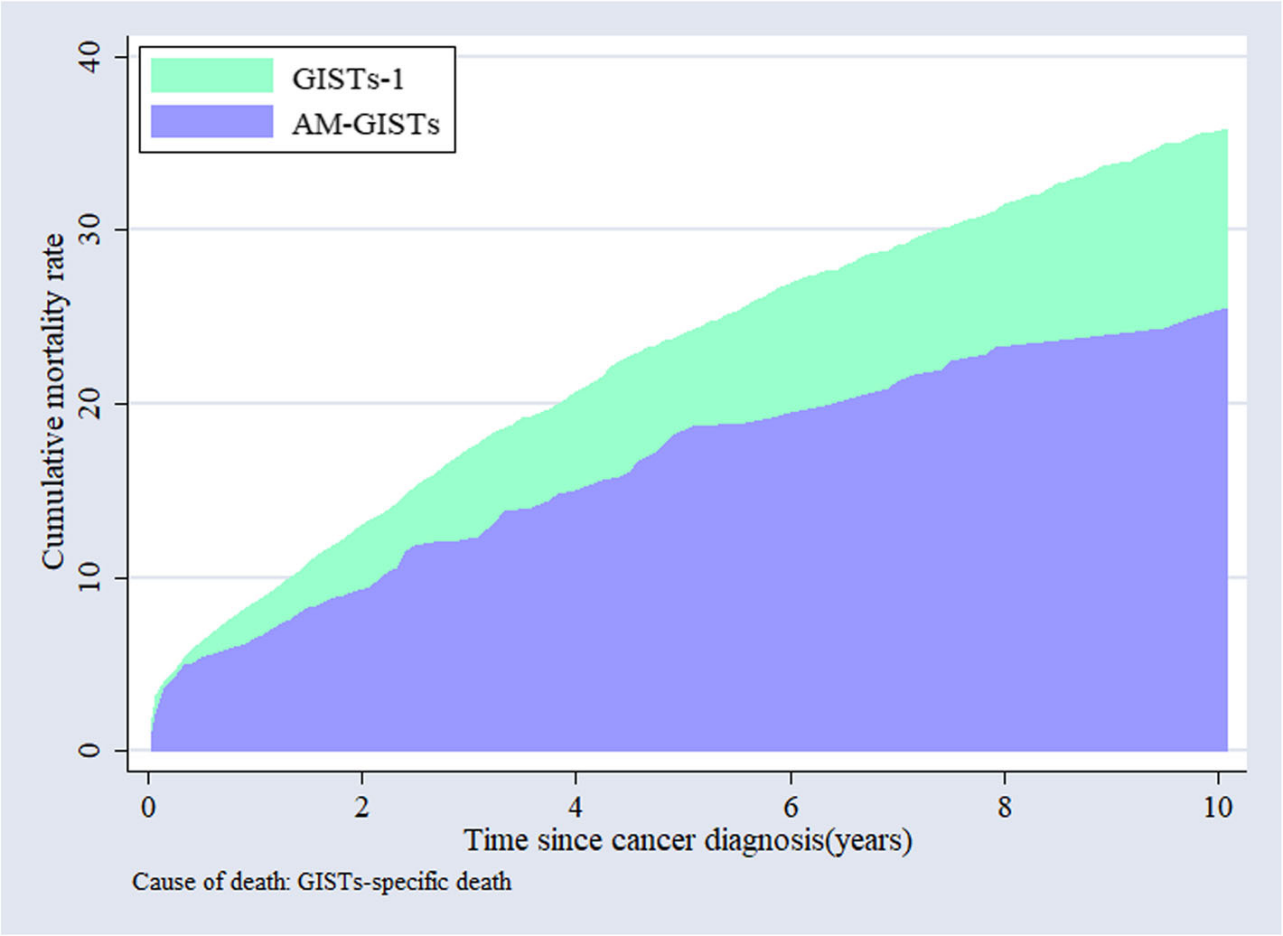
Cumulative mortality rate of GISTs-specific death among patients with GISTs-1 and AM-GISTs

Table 3 and Fig. 3a, b summarize the unadjusted OS and GISTs-specific survival of AM-GISTs compared with GISTs-1. The patients with AM-GISTs exhibited a worse prognosis than GIST-1 (1-year OS, 84.8% vs. 89.0%; 3-year OS, 70.7% vs. 77.7%; 5-year OS, 59.0% vs. 68.7%; 10-year OS, 40.3% vs. 50.0%, respectively, *p* < 0.001), while the GISTs-specific survival rate of AM-GISTs was significantly superior to that of the corresponding GISTs-1 group (*p* = 0.002). Patients with AM-GISTs had a lower cumulative rate of GISTs-specific mortality than their GISTs-1 counterparts (Fig. 4). Moreover, we further attempted to explore the OS and GISTs-specific survival for those with AM-GISTs, which was grouped by the first primary malignancy sites (*n* = 851, Fig. 3c, d). Survivors with breast cancer demonstrated a better OS than that of the prostate, urinary system, and others (*p* = 0.043, *p* = 0.004, and *p* = 0.007; respectively, Fig. 3c). As for GISTs-specific survival, patients with carcinoma of the large intestine were superior to that of prostate and urinary system (*p* = 0.03, *p* = 0.017; respectively, Fig. 3d), with the corresponding percentages of 8.7% vs. 20.3% and 20.3% of patients dying of GISTs, respectively. Additionally, no significant differences were observed between other groups.

**Table 3:** Comparison of Kaplan-Meier OS and GISTs-specific survival probabilities: patients with gastrointestinal stromal tumors after another malignancy (AM-GISTs) compared with GISTs only (GISTs-1)

	AM-GISTs (*n* = 851)	GISTs-1 (*n* = 7660)	p*
**OS**			**< 0.001**
1 year	84.8	89.0	
3 years	70.7	77.7	
5 years	59.0	68.7	
10 years	40.3	50.0	
**GISTs-specific survival**			**0.002**
1 year	93.4	91.5	
3 years	86.8	82.2	
5 years	79.1	75.1	
10 years	68.9	61.8	

*GISTs* gastrointestinal stromal tumors, *OS* overall survival

*Comparison between AM-GISTs and GISTs-1 group using by a 2-tailed log-rank test

### Prognostic factors affecting OS and GISTs-specific survival

Table 4 describes the univariate and multivariate analyses of prognostic factors that could potentially affect OS and GISTs-specific survival in this cohort. Bring the significant factors identified by univariate analysis into the Cox multivariate regression proportional hazards model, we found that patients with a history of previous another malignancy demonstrated as an adverse prognostic factor for OS when compared with those diagnosed with GISTs only (hazard ratio [HR], 1.11; 95% confidence interval [95% CI], 0.99–1.25), albeit this did not reach statistical significance (*p* = 0.085). Specifically, the risk of death increased significantly in patients with a previous malignancy of hematological (*p* = 0.014) and reproductive system (*p* = 0.002) in comparison to GISTs-1 (Table 5). Moreover, males, advanced age, increased tumor size, those with small intestine GISTs, tumors with moderately/poorly differentiated, and distant condition were also significant adverse risk factors for OS. As for GISTs-specific survival, patients with AM-GISTs had a considerably lower risk of dying from GISTs compared to that of GISTs-1 (HR, 0.71; 95% CI, 0.59–0.84; *p* < 0.001), particularly for those with antecedent prostate cancer (HR, 0.68; 95% CI, 0.51–0.91; *p* = 0.009) and melanoma of the skin diagnosis (HR, 0.33; 95% CI, 0.15–0.75; *p* = 0.007).

**Table 4:** Univariate and multivariate analysis of factors associated with overall and GISTs-specific survival using Cox proportional hazards regression modeling

	Overall survival				GISTs-specific survival			
	Univariate		Multivariate*		Univariate		Multivariate*	
Covariates	HR (95% CI)	*p*	HR (95% CI)	*p*	HR (95% CI)	*p*	HR (95% CI)	*p*
**History of previous malignancy**								
No (GISTs-1)	Reference		Reference		Reference		Reference	
Yes (AM-GISTs)	1.37 (1.22–1.53)	**< 0.001**	1.11 (0.99–1.25)	**0.085**	0.76 (0.64–0.90)	**0.002**	0.71 (0.59–0.84)	**< 0.001**
**Age at diagnosis, years**								
18–35	Reference		Reference		Reference		Reference	
36–50	1.26 (0.97–1.65)	**0.084**	1.26 (0.96–1.64)	**0.091**	1.20 (0.91–1.59)	**0.192**	1.21 (0.92–1.60)	**0.181**
51–65	1.70 (1.32–2.19)	**< 0.001**	1.88 (1.46–2.43)	**< 0.001**	1.43 (1.10–1.87)	**0.008**	1.66 (1.27–2.17)	**< 0.001**
66–80	2.94 (2.29–3.78)	**< 0.001**	3.28 (2.54–4.24)	**< 0.001**	1.90 (1.46–2.48)	**< 0.001**	2.29 (1.75–3.01)	**< 0.001**
≥ 80	6.47 (5.01–8.35)	**< 0.001**	6.71 (5.16–8.72)	**< 0.001**	3.68 (2.80–4.85)	**< 0.001**	4.19 (3.15–5.57)	**< 0.001**
**Sex**								
Male	Reference		Reference		Reference		Reference	
Female	0.78 (0.73–0.84)	**< 0.001**	0.71 (0.66–0.77)	**< 0.001**	0.77 (0.70–0.84)	**< 0.001**	0.76 (0.69–0.83)	**< 0.001**
**Race/ethnicity**								
White	Reference		Reference		Reference		Reference	
Black	1.14 (1.05–1.25)	**0.003**	1.08 (0.98–1.19)	**0.108**	1.14 (1.02–1.27)	**0.018**	1.03 (0.92–1.16)	**0.628**
Other	0.83 (0.74–0.92)	**0.001**	0.91 (0.81–1.02)	**0.110**	0.85 (0.75–0.97)	**0.018**	0.91 (0.79–1.04)	**0.169**
**Marital status**								
Single	Reference		Reference		Reference		Reference	
Married/domestic partner	0.84 (0.76–0.93)	**0.001**	0.74 (0.67–0.82)	**< 0.001**	0.79 (0.70–0.88)	**< 0.001**	0.76 (0.67–0.85)	**< 0.001**
Widowed/separated/divorced	1.44 (1.29–1.61)	**< 0.001**	0.99 (0.89–1.12)	**0.956**	1.20 (1.06–1.37)	**0.006**	0.97 (0.84–1.11)	**0.631**
Unknown	0.84 (0.69–1.02)	**0.075**	0.71 (0.58–0.86)	**0.001**	0.71 (0.56–0.90)	**0.005**	0.66 (0.52–0.85)	**0.001**
**Primary site**								
Stomach	Reference		Reference		Reference		Reference	
Small intestine	1.02 (0.94–1.11)	**0.698**	1.12 (1.02–1.22)	**0.015**	1.12 (1.01–1.24)	**0.031**	1.13 (1.01–1.25)	**0.029**
Other	1.70 (1.55–1.87)	**< 0.001**	1.07 (0.97–1.18)	**0.170**	2.06 (1.85–2.30)	**< 0.001**	1.11 (0.99–1.25)	**0.075**
**Lymph node metastasis**								
No	Reference		Reference		Reference		Reference	
Yes	1.56 (1.26–1.94)	**< 0.001**	0.97 (0.77–1.21)	**0.771**	1.69 (1.32–2.17)	**< 0.001**	0.93 (0.72–1.20)	**0.582**
Unknown	1.26 (1.15–1.37)	**< 0.001**	0.94 (0.85–1.03)	**0.169**	1.18 (1.06–1.31)	**0.002**	0.82 (0.74–0.92)	**0.001**
**Tumor size, cm**								
0–2	Reference		Reference		Reference		Reference	
> 2 to ≤ 5	0.83 (0.69–1.01)	**0.059**	0.69 (0.57–0.84)	**< 0.001**	1.05 (0.78–1.42)	**0.737**	0.84 (0.62–1.14)	**0.265**
> 5 to ≤ 10	1.32 (1.10–1.57)	**0.003**	1.04 (0.87–1.25)	**0.648**	2.28 (1.72–3.01)	**< 0.001**	1.59 (1.20–2.10)	**0.001**
> 10	2.01 (1.68–2.40)	**< 0.001**	1.30 (1.08–1.57)	**0.005**	4.03 (3.06–5.31)	**< 0.001**	2.10 (1.58–2.78)	**< 0.001**
Unknown	2.92 (2.44–3.50)	**< 0.001**	1.40 (1.16–1.70)	**0.001**	5.95 (4.50–7.87)	**< 0.001**	2.22 (1.66–2.96)	**< 0.001**
**Grade**								
Well differentiated	Reference		Reference		Reference		Reference	
Moderately differentiated	1.52 (1.23–1.88)	**< 0.001**	1.27 (1.03–1.58)	**0.027**	2.12 (1.58–2.86)	**< 0.001**	1.64 (1.21–2.21)	**0.001**
Poorly differentiated	3.31 (2.64–4.15)	**< 0.001**	2.18 (1.73–2.74)	**< 0.001**	5.90 (4.38–7.97)	**< 0.001**	3.24 (2.40–4.39)	**< 0.001**
Undifferentiated	3.99 (3.26–4.90)	**< 0.001**	2.48 (2.02–3.05)	**< 0.001**	7.05 (5.33–9.32)	**< 0.001**	3.56 (2.68–4.72)	**< 0.001**
Unknown	2.35 (1.98–2.79)	**< 0.001**	1.48 (1.24–1.76)	**< 0.001**	3.60 (2.79–4.63)	**< 0.001**	1.94 (1.50–2.51)	**< 0.001**
**SEER stages**								
Localized	Reference		Reference		Reference		Reference	
Regional	1.87 (1.68–2.08)	**< 0.001**	1.58 (1.41–1.77)	**< 0.001**	2.58 (2.26–2.94)	**< 0.001**	1.84 (1.59–2.12)	**< 0.001**
Distant	3.72 (3.43–4.04)	**< 0.001**	2.41 (2.18–2.66)	**< 0.001**	5.97 (5.38–6.61)	**< 0.001**	3.23 (2.85–3.65)	**< 0.001**
Unstaged/unknown	2.05 (1.81–2.33)	**< 0.001**	1.30 (1.13–1.49)	**< 0.001**	2.64 (2.26–3.09)	**< 0.001**	1.54 (1.30–1.84)	**< 0.001**

*GISTs* gastrointestinal stromal tumors, *CI* confidence interval, *SEER* the Surveillance, Epidemiology, and End Results database

*HR was additionally adjusted for education, median household income, year of diagnosis, chemotherapy, and surgery of primary

**Table 5:** Hazard ratios of overall survival and GISTs-specific survival among patients with AM-GISTs, stratified by sites of the first malignancy, compared with those with GISTs only

	Overall survival*		GISTs-specific survival*	
Covariates	HR (95% CI)	*p*	HR (95% CI)	*p*
**GISTs-1**	Reference		Reference	
**AM-GISTs**				
By sites of the first malignancy				
Prostate	0.87 (0.70–1.07)	**0.179**	0.68 (0.51–0.91)	**0.009**
Breast	0.90 (0.66–1.21)	**0.469**	0.79 (0.53–1.17)	**0.237**
Large intestine	1.24 (0.90–1.71)	**0.191**	0.52 (0.27–0.99)	**0.050**
Urinary system	1.24 (0.88–1.76)	**0.224**	0.85 (0.51–1.42)	**0.535**
Hematological system	1.66 (1.11–2.49)	**0.014**	1.25 (0.69–2.27)	**0.455**
Reproductive system	1.85 (1.25–2.73)	**0.002**	0.95 (0.49–1.84)	**0.877**
Melanoma of the skin	0.75 (0.49–1.16)	**0.192**	0.33 (0.15–0.75)	**0.007**
Others	1.76 (1.34–2.30)	**< 0.001**	0.67 (0.40–1.14)	**0.143**

*GISTs* gastrointestinal stromal tumors, *CI* confidence interval

*HR was adjusted for age, sex, race, marital status, education, median household income, year of diagnosis, primary site, chemotherapy, surgery of primary, lymph node metastasis, tumor size, grade, and SEER stages

## Discussion

To the best of our knowledge, this is the first study to comprehensively describe prognostic features and outcomes for patients with GISTs after another malignancy using a large population-based cohort. Based on 851 patients with AM-GISTs and 7660 patients with GISTs-1, an important new finding in the present study was the significantly superior GISTs-specific survival among patients with GISTs after another malignancy compared with those who had GISTs only. To some extent, this perhaps reflecting heightened surveillance in AM-GISTs group patients compared with the general population. In addition, GISTs in smaller size and with less aggressive in the AM-GISTs group may be the reason for its GISTs-specific survival improvement. An antecedent another malignancy diagnosis, however, is associated with a relatively worse overall survival. Moreover, we found that prostate cancer, followed by breast cancer, carcinoma of the large intestine, and malignant tumor of the urinary system were the most common first primary malignancy among patients with AM-GISTs.

### Clinicopathologic characteristics

Although a wealth of research suggests that GISTs patients are twice as likely to have a risk of developing a second neoplasm as the general population [15, 22], GISTs as a second primary malignancy is rarely reported. The most common GISTs-associated malignancies, either synchronously or metachronously, were gastrointestinal carcinomas (47%) and prostate cancer (9%) [16]. In contrast, we found that breast and prostate cancers were the most common first primary malignancy in this study, with a similar conclusion to Hechtman et al. [20] and Pandurengan et al. [18]. The biological plausibility that links the history of another malignancy of different origins to GISTs is currently unclear. It is important to note that an activating mutation of *KIT*, which is considered to be key drivers of GISTs molecular pathogenesis, could be implicated both in solid and other malignancies [23]. The previous study showed that GISTs and renal cell carcinoma might occur as familial tumors related to a mutation in *KIT* [17]. In addition, Hechtman et al. have reported that seminoma and melanomas, which were diagnosed before GISTs, both harbored *KIT* mutation [20], which is also the primary pathogenesis for GISTs. Due to the nature of the SEER data, however, we cannot further explore this issue. As such, the correlation between a previous malignancy and GISTs should be determined in the future. However, molecular assessment of the first primary malignancy may be valuable in predicting the occurrence of the second primary GISTs. AM-GISTs tended to be diagnosed at an early stage and have a smaller size at the time of GISTs diagnosis compared with GISTs-1. It is plausible that patients in the AM-GISTs group may, by virtue of their concerns about the first cancer recurrence/metastasis, be more likely to undergo diagnostic workups for related symptoms, which in turn leading to early diagnosis of GISTs in the present study. Previous studies have documented that localized/regional non-small-cell lung cancer (NSCLC) occurs mostly within 10 years after diagnosis of the first primary cancer [11, 13]. In line with their findings, it is noted that 58.3% and 79.2% of AM-GISTs were diagnosed ≤ 5 and ≤ 10 years after another malignancy in this cohort, respectively. Moreover, this is the first study demonstrating that AM-GISTs patients were more likely to be diagnosed at an older age at the time of GISTs diagnosis and diagnosed in relatively later decades compared with GISTs-1 patients, respectively.

### Survival outcomes

Up till now, there is limited information on the prognosis for patients with GISTs after another malignancy available. Patients with GISTs without any other primary appeared to have a trend toward the better OS compared with those with a first primary and subsequent GISTs [18]. In other similar studies, but not for GISTs, it showed that a history of chronic lymphocytic leukemia and HL portends a significantly worse overall survival for patients diagnosed with NSCLC [11, 24], while patients with an antecedent breast cancer diagnosis does not affect the prognosis [13]. In this study, patients with AM-GISTs appeared to have an inferior OS compared to that of GISTs-1, particularly in patients with a previous malignancy of hematological and reproductive system. Not surprisingly, due in part to other malignancy deaths, the overall survival of AM-GISTs versus GISTs-1 was relatively poorer, but the extent of the difference was modest in our study. On the contrary, our finding showed that GISTs-specific survival was significantly superior among patients with antecedent another malignancy compared with GISTs only (HR = 0.71; 95% CI, 0.59–0.84; *p* < 0.001), especially for those with antecedent prostate cancer and melanoma of the skin diagnosis. Theories to explain this phenomenon including that the majority of AM-GISTs patients diagnosed at an early stage and with a relatively smaller size, as well as more aggressive treatments, may be adopted for GISTs. Moreover, the favorable cancer-specific survival was limited to the first 5 years after diagnosis for those diagnosed as lung cancer after another malignancy, but the survival benefits were somewhat attenuated in the long run [25]. Taken together, we believe that it is still indispensable to increasing surveillance for these patients.

### Causes of deaths

It is worth noting that we observed a very high mortality rate associated with second primary malignancies, with > 40% of patients died of their second malignancy and only 26% of patients died from their first primary cancer in the cohort of AM-GISTs. By comparison, a similar study demonstrated that up to 85% of patients died of NSCLC in the regional/distant NSCLS after HL diagnosis, and only 4% of all patients with HL-NSCLC died of HL [11]. Theoretically, this may be partially attributed to more favorable outcomes for their first malignancy as compared second primary tumors. A study based on pooled populations showed that most GISTs recurrences took place within the first 5 years of follow-up, thereby affecting long-term outcomes, with estimated 5- and 10-year OS of 72.3% and 56.4%, respectively [26]. It is important to note that heart/cardiovascular disease merely accounting for a small number of deaths in the AM-GISTs and GISTs-1 group, with the corresponding percentages of 11.2% and 9.9%, respectively, which is similar to the conclusions of Milano et al. [13].

### Treatments for GISTs

Primary tumor resection with negative margins is the principal curative option for GISTs. In AM-GISTs and GISTs-1 group, the distribution of the proportion of GISTs-directed surgery was comparable, with 75.3% and 77.3% of patients underwent surgery, respectively. Moreover, routine lymphadenectomy is not recommended, because of the low incidence of lymphatic metastasis [27, 28], and its prognostic value also remains controversial [29, 30]. In our study, we found that lymph node metastasis was not associated with a worse prognosis. Interestingly, the GISTs-1 group was more likely to undergo chemotherapy, but our study failed to address this issue in-depth because of no detailed chemotherapy regimens available in SEER database.

### Strengths and limitations

The primary strength of the current study is that the sizeable number of patients (*n* = 8511) were identified in a population-based setting. To a certain extent, it ensures the minimal common biases, which is strengthening the generalizability of our result. In addition, with such a large number of patients and long-term follow-up, it also allowed for analyses of outcomes based on clinicopathologic characteristics and cancer-related variables. Limitations of registry-based studies [27, 31], due to the nature of the SEER database, include lack of detail information about chemotherapy, whether or not tyrosine kinase inhibitors used, mutation status, vascular invasion, tumor rupture or not, as well as surgical margin status. In addition, the mitotic count was systematically recorded after 2009; thus, it is not included in the present study due to many missing values. Finally, it should be borne in mind that SEER program registry is primarily selected to represent the US population, and the results may not apply to other populations or countries.

## Conclusions

Patients with second primary GISTs have a favorable GISTs-specific survival in contrast to those with GISTs only, while the OS is slightly compromised in AM-GISTs patients. In the cohort of AM-GISTs, most patients (> 40%) died of their second malignancy, and only 26% of patients died from their first primary cancer. With the increasing number of AM-GISTs diagnosed in recent years, it is necessary to explore effective surveillance and treatment strategies in this population.

## Data Availability

The data that support the findings of this study are available from SEER registry but restrictions apply to the availability of these data, which were used under license for the current study, and so are not publicly available.

## Abbreviations

SEER: Surveillance, Epidemiology, and End Results
GISTs: Gastrointestinal stromal tumors
OS: Overall survival
GSS: GISTs-specific survival
HR: Hazard ratio
HL: Hodgkin lymphoma
NSCLC: Non-small-cell lung cancer
